# Socially isolated individuals are more prone to have newly diagnosed and prevalent type 2 diabetes mellitus - the Maastricht study –

**DOI:** 10.1186/s12889-017-4948-6

**Published:** 2017-12-19

**Authors:** Stephanie Brinkhues, Nicole H. T. M. Dukers-Muijrers, Christian J. P. A. Hoebe, Carla J. H. van der Kallen, Pieter C. Dagnelie, Annemarie Koster, Ronald M. A. Henry, Simone J. S. Sep, Nicolaas C. Schaper, Coen D. A. Stehouwer, Hans Bosma, Paul H. M. Savelkoul, Miranda T. Schram

**Affiliations:** 1 Department of Medical Microbiology, Maastricht University Medical Centre, P.O. Box 5800, 6202 AZ Maastricht, the Netherlands; 2 Department of Sexual Health, Infectious Diseases and Environmental Health, Public Health Service South Limburg, P.O. Box 33, 6400 AA Heerlen, the Netherlands; 3 CAPHRI Care and Public Health Research Institute, Maastricht University, P.O. Box 616, 6200 MD Maastricht, the Netherlands; 4 Department of Medicine, Maastricht University Medical Centre, P.O. Box 5800, 6202 AZ Maastricht, the Netherlands; 5 CARIM Cardiovascular Research Institute Maastricht, Maastricht University, P.O. Box 616, 6200 MD Maastricht, the Netherlands; 6 Department of Epidemiology, Maastricht University, P.O. Box 616, 6200 MD Maastricht, the Netherlands; 7 Department of Social Medicine, Maastricht University, P.O. Box 616, 6200 MD Maastricht, the Netherlands; 8 Department of Medical Microbiology & Infection Control, VU University Medical Centre, P.O. Box 7057, 1007 MB Amsterdam, the Netherlands; 9 Heart and Vascular Centre, Maastricht University Medical Centre, P.O. Box 5800, 6202 AZ Maastricht, the Netherlands

**Keywords:** Type 2 diabetes, Pre-diabetes, Social network, Social support, Prevention

## Abstract

**Background:**

Social isolation is associated with type 2 diabetes (T2DM), but it is unclear which elements play a crucial role in this association. Therefore, we assessed the associations of a broad range of structural and functional social network characteristics with normal glucose metabolism, pre-diabetes, newly diagnosed T2DM and previously diagnosed T2DM.

**Methods:**

Participants originated from The Maastricht Study, a population-based cohort study (*n* = 2861, mean age 60.0 ± 8.2 years, 49% female, 28.8% T2DM (oversampled)). Social network characteristics were assessed through a name generator questionnaire. Diabetes status was determined by an oral glucose tolerance test. We used multinomial regression analyses to investigate the associations between social network characteristics and diabetes status, stratified by sex.

**Results:**

More socially isolated individuals (smaller social network size) more frequently had newly diagnosed and previously diagnosed T2DM, while this association was not observed with pre-diabetes. In women, proximity and the type of relationship was associated with newly diagnosed and previously diagnosed T2DM. A lack of social participation was associated with pre-diabetes as well as with previously diagnosed T2DM in women, and with previously diagnosed T2DM in men. Living alone was associated with higher odds of previously diagnosed T2DM in men, but not in women. Less emotional support related to important decisions, less practical support related to jobs, and less practical support for sickness were associated with newly diagnosed and previously diagnosed T2DM in men and women, but not in pre-diabetes.

**Conclusion:**

This study shows that several aspects of structural and functional characteristics of the social network were associated with newly and previously diagnosed T2DM, partially different for men and women. These results may provide useful targets for T2DM prevention efforts.

**Electronic supplementary material:**

The online version of this article (10.1186/s12889-017-4948-6) contains supplementary material, which is available to authorized users.

## Background

The growing number of people with chronic conditions, such as type 2 diabetes mellitus (T2DM), is a rising problem in health care. An estimated 171 million individuals worldwide had T2DM in 2000, and this number is expected to increase to 366 million individuals in 2030, with a higher prevalence in men [1]. Because T2DM leads to severe complications and significantly reduces life expectancy [2], and multiborbidity is common [3], these figures underline the need for interventions that can prevent the development of T2DM. Several environmental and lifestyle factors, as well as psychosocial factors such as depression and stress, have been identified as relevant for the development of T2DM [4–7]. Recently, there is raising interest for the role of social network characteristics in the development of T2DM [7–14]. Prevention strategies that promote social integration and participation may prove promising [15–18]. Among individuals with T2DM, beneficial effects of social support have been reported on diabetes care [19], activation for self-management [20], and health/health-related behaviors [21].

Given the results of previous research, a more detailed and conjoint investigation of a broad range of social network characteristics is essential. Previous studies on social network characteristics have typically focused on either structural or functional characteristics, while both have been found to associate with T2DM risk [7–14]. For example, the single indicator low emotional support is associated with a doubled risk of T2DM in women [7], while prevalent T2DM is also related to lower emotional support [8]. Negative friend support increases the odds of T2DM by 30% in both men and women [14]. In addition, poor structural support has been shown to increase the risk of T2DM in men by 50%, particularly evident among those with a low education level [9]. Furthermore, several studies have found that living alone was an independent predictor of T2DM in men, but not in women [7, 11, 12]. In contrast, one study reported that high social integration increased the odds of T2DM in men [12]. However, the associations of social network characteristics with pre-diabetes or newly diagnosed T2DM were less clear, studies accounting for pre-diabetes and newly diagnosed T2DM are rare [12, 13].

In light of these considerations, the aim of the present study was to assess the associations of a broad range of social network characteristics with diabetes status. Specifically, we assessed whether structural characteristics such as social network size, contact frequency, type of relationship, living alone and social participation are associated with pre-diabetes and newly diagnosed and previously diagnosed T2DM. Next, we addressed the question of whether functional characteristics of the social network (social support) are associated with pre-diabetes, newly diagnosed T2DM and previously diagnosed T2DM. To investigate the differences between men and women, all analyses were stratified by sex.

## Methods

### Study population

We used data from The Maastricht Study, an observational prospective population-based cohort study. The rationale and methodology have been described previously [22]. In brief, the study focuses on the etiology, pathophysiology, complications and comorbidities of type 2 diabetes mellitus (T2DM) and is characterized by an extensive phenotyping approach. The study uses state-of-the-art imaging techniques and extensive biobanking to determine both determinants and clinical outcomes of health status.

Eligible for participation were all individuals aged between 40 and 75 years and living in the southern part of the Netherlands. Participants with and without diabetes were recruited through mass media campaigns and from the municipal registries and the regional Diabetes Patient Registry via mailings. Recruitment was stratified according to known T2DM status, with an oversampling of individuals with T2DM, for reasons of efficiency. Enrollment started in November 2010 and is still ongoing, aiming to include 10.000 participants. The present report includes cross-sectional data from the first 3451 participants, who completed the baseline survey between November 2010 and September 2013. The examinations of each participant were performed within a time window of 3 months. Further information on The Maastricht study can be found elsewhere [22].

After excluding participants who did not provide data on their social network (*n* = 447 (12.9%), the main reason for missing data was incomplete questionnaires), participants with type 1 diabetes (*n* = 33), and other types of diabetes (n = 4), and participants with missing information on covariates (*n* = 106), a total of 2861 participants were included in the present analyses. The participants without social network data did not differ from those with these data with respect to diabetes status, sex, educational level, or body mass index (BMI). However, the participants who did not provide social network data were slightly younger than those who did (mean age 59 versus 60 years, (*p* < 0.001)).

### Measurements

#### Glucose metabolism status

To determine glucose metabolism status, all participants (except those who used insulin) underwent a standardized 75 g oral glucose tolerance test (OGTT) after an overnight fast [22]. Glucose metabolism was defined according to the World Health Organization 2006 criteria as normal glucose metabolism (NGM), impaired fasting glucose (IFG), impaired glucose tolerance (IGT), or T2DM [23]. Individuals on diabetes medication were classified as having T2DM. We defined pre-diabetes as having either IFG or IGT and newly diagnosed (unaware) T2DM as negative self-reported T2DM with a positive OGTT.

#### Social network questionnaire

Data on individual social networks were collected through a questionnaire using a name generator method [24, 25]. A detailed description of this questionnaire can be found in the additional file (see Additional file 1). The name generator first requires a respondent to identify actual persons, and then several additional questions about these individuals are asked (sex, age, type of relationship, geographic distance, and the number of members who provided informational, practical or emotional support).

#### Structural characteristics of the social network

The structural network characteristics were computed from the questionnaire. In brief, network size was defined as the total number of unique network members (alters) mentioned in the questionnaire. Total contacts per half year was defined as the sum of all contacts per half year. In addition, the percentage of network members that the participant (ego) had daily/weekly contact with, that were household members, that lived within walking distance, and the percentage of network members that were family members or friends was computed. Those social network constructs of percentages within the network were defined in steps of 10%. Based on an average network size of 10 network members, a change in one network member corresponds to 10%.

Living alone was defined as a person who lived alone in his household. Social participation was defined as membership in, for instance, a sports club, religious group, volunteer organization, discussion group, self-support group, internet club, or other organization. Additional information on structural social network characteristics used in the present study can be found in Table 1.

**Table 1:** Variable descriptions of the structural and functional social network characteristics

Variable name	Definition	Unit of measurement (possible range)
Structural characteristics of the social network		
Network size	The total number of unique network members mentioned in the questionnaire. Participants with a smaller social network size were considered as more socially isolated.	N (0–40)
*Contact frequency*		
Total contacts per half year	A contact was defined as an interaction between persons. Total contacts (interactions between persons) per half year were computed as follows. We used the highest contact frequency (e.g., daily contact) for every network member as an indicator of the actual contact frequency. Second, we recoded the answer categories of the questionnaire to an estimated number of contacts per half year. For example, “half-yearly” was assumed to comprise one contact, “quarterly” two contacts, “monthly” 6 contacts and “daily or weekly” 48 contacts. Third, we computed the sum of all contacts per half year as the total contact frequency.	N (0–1920)
Percentage of daily-weekly contact	We calculated the percentage of network members that the participant had daily or weekly contact with as the number of daily/weekly contacts divided by network size.	% (0–100)
*Proximity*		
Percentage of network members living within walking distance	We considered geographic proximity as the percentage of all network members who lived within walking distance, calculated as the number of network members living within walking distance divided by network size.	% (0–100)
*Type of relationship*		
Percentage household members	We calculated the percentage of household members as the number of network members living in the same household divided by network size.	% (0–100)
Percentage family members	We calculated the percentage of family members within the network as the number of family members divided by the network size.	% (0–100)
Percentage friends	We calculated the percentage of friends within the network as the number of friends divided by the network size.	% (0–100)
Living alone	Living alone was defined as a person who lived alone in his/ her household.	(yes/no)
Social participation	Social participation was defined as membership in, for instance, a sports club, religious group, volunteer organization, discussion group, self-support group, internet club, or other organization.	(yes/no)
Functional characteristics of the social network		
Informational support	Informational support was defined as the number of network members that give advice on problems	N (0–5)
Emotional support (discomfort)	Emotional support related to discomfort was defined as the number of network members that provide emotional support when participants were feeling unwell	N (0–5)
Emotional support (important decisions)	Emotional support related to important decisions was defined as the number of network members that provide the opportunity to discuss important matters	N (0–5)
Practical support (jobs)	Practical support related to jobs was defined as the number of network members that help with small and larger jobs around the house	N (0–5)
Practical support (sickness)	Practical support related to sickness was defined as the number of network members that provide practical help when participants were sick	N (0–5)

#### Functional characteristics of the social network (social support)

Participants were asked to indicate the number of members who provided informational support, emotional support related to discomfort, emotional support related to important decisions, practical support related to jobs, and practical support related to sickness. For every type of support, participants could name a maximum of 5 network members. This results in a possible range of 0 to 5 for the functional characteristics of the social network. Additional information on functional social network characteristics used in the present study can be found in Table 1.

#### General measurements

Self-administered questionnaires were used to assess educational level, employment status, smoking status, alcohol consumption, history of cardiovascular disease (CVD), diabetes medication use and diabetes duration. Body mass index (BMI) and hypertension were measured at the study centre [22]. General health was assessed with the SF-36 Health Survey and transformed scale scores were calculated according to Ware et al. (1994) [26].

### Statistical analysis

Descriptive analyses were performed to examine the characteristics of the study population, and the results were presented as the mean and standard deviation (SD) or percentages and numbers. To assess the differences between participants with NGM, pre-diabetes, newly diagnosed T2DM and previously diagnosed T2DM, we performed chi-square, analysis of variance (ANOVA) and Kruskal-Wallis tests, as appropriate. We conducted multinomial logistic regression analyses to examine the association of the social network variables with diabetes status, using NGM as reference. For every network variable, odds ratios (ORs) and 95% confidence intervals (95%CIs) were reported. For descriptive purposes, social network variables were reversed, i.e., multiplied by −1 (lower values on social network variables indicated risk factor). Every network variable was assessed separately, risk estimates were adjusted for age, BMI, educational level, employment status, alcohol consumption, smoking status, hypertension, prior CVD and general health status (SF36). As previous research has shown different associations between social network and diabetes status between men and women [7, 9, 11, 12], we tested for statistical interactions (effect modification) of the network variables with sex. Because the majority of the social network variables showed an interaction with sex (*p* < 0.1), all analyses were stratified by sex. All analyses were conducted using IBM SPSS software version 21.0 (IBM Corp. Armonk, NY, USA). Associations with *p* ≤ 0.05 were considered statistically significant.

## Results

The overall study population consisted of 2861 participants with a mean age of 60.0 ± 8.2 years, of whom slightly less than half were women (49%). Table 2 presents descriptive characteristics according to diabetes status. A total of 1623 (56.7%) participants had a normal glucose metabolism status (NGM), 430 (15.0%) had pre-diabetes, 111 (3.9%) were newly diagnosed as T2DM at study entry, and 697 (24.4%) had previously diagnosed T2DM. Participants with T2DM were older, more often men, had a higher BMI, were lower educated, were more often retired, were more often current smokers, were less often high alcohol consumers, and had prior CVD and hypertension more often than participants with NGM or pre-diabetes. In participants with previously diagnosed T2DM, the median self-reported diabetes duration was 7 years (IQR 3.0–12.0). Participants with newly diagnosed T2DM were more often higher educated, less often obese, less often current smokers, more often high alcohol consumers and had prior CVD and hypertension less often than participants with previously diagnosed T2DM.

**Table 2:** General and social network characteristics of the study population

	NGM (*n* = 1623)	Pre-diabetes (*n* = 430)	Newly diagnosed T2DM (*n* = 111)	Previously diagnosed T2DM (*n* = 697)	*P*-value^1^
General measurements					
Age	58.1 ± 8.1	61.6 ± 7.5	62.9 ± 7.5	62.7 ± 7.7	<0.001
Male sex (%)	42.2	53.3	63.1	69.4	<0.001
Body mass index (kg/m^2^)	25.5 ± 3.6	27.7 ± 4.3	28.8 ± 4.8	29.9 ± 5.0	
*Educational level* (%)					
- low^2^	26.1	34.7	34.2	47.1	<0.001
- intermediate^3^	27.5	28.1	30.6	27.7	
- high^4^	45.7	36.3	34.2	24.5	
*Employment status* (%)					
- employed	46.8	35.3	28.8	27.8	<0.001
- retired	26.6	36.0	45.0	37.0	
- no paid job	19.6	19.8	19.8	20.1	
- not known	7.1	8.8	6.3	15.1	
*Smoking status* (%)					
- never	39.6	29.8	33.3	27.7	<0.001
- former	48.4	57.0	57.7	55.8	
- current	11.9	13.3	8.1	16.1	
Alcohol consumption, glasses per week	7.3 ± 7.1	9.1 ± 10.6	9.3 ± 10.6	6.1 ± 8.5	<0.001
Prior CVD (%)	11.6	12.1	20.7	27.5	<0.001
Hypertension (%)	41.2	63.6	75.7	83.9	<0.001
Diabetes medication use (%)	n/a	n/a	n/a	90.9	n/a
Diabetes duration (years; median, Q1-Q3; *n* = 567)	n/a	n/a	n/a	7.0 (3.0–12.0)	n/a
Structural characteristics of the social network					
Network size	11.00 ± 5.15	10.02 ± 5.08	7.68 ± 4.59	7.61 ± 4.38	<0.001
*Contact frequency*					
Total contacts per half year	249.33 ± 144.09	233.13 ± 145.26	193.14 ± 123.39	196.55 ± 125.58	<0.001
Percentage of daily-weekly contact	46.29 ± 24.41	47.15 ± 25.09	53.67 ± 28.04	54.16 ± 28.01	<0.001
*Proximity*					
Percentage of network members living within walking distance	28.96 ± 21.28	30.79 ± 23.60	27.51 ± 24.07	27.67 ± 24.20	0.158
*Type of relationship*					
Percentage household members	14.00 ± 12.48	14.42 ± 13.84	21.19 ± 20.71	17.53 ± 17.41	<0.001
Percentage family members	55.94 ± 22.34	58.30 ± 23.68	61.78 ± 27.22	64.68 ± 26.00	<0.001
Percentage friends	30.05 ± 20.30	27.23 ± 20.95	22.76 ± 21.06	21.43 ± 21.96	<0.001
Living alone (%)	14.7	17.4	17.1	20.2	<0.05
Social participation (%)	71.6	64.2	61.1	56.4	<0.001
Functional characteristics of the social network					
Informational support^a^	3.5 ± 1.6	3.2 ± 1.7	2.7 ± 1.7	2.7 ± 1.7	<0.001
Emotional support (discomfort) ^a^	3.0 ± 1.6	2.6 ± 1.6	2.1 ± 1.5	2.2 ± 1.5	<0.001
Emotional support (important decisions) ^a^	3.4 ± 1.5	2.9 ± 1.6	2.5 ± 1.7	2.4 ± 1.5	<0.001
Practical support (jobs)^a^	3.0 ± 1.5	2.7 ± 1.5	2.3 ± 1.4	2.4 ± 1.4	<0.001
Practical support (sickness) ^a^	2.5 ± 1.4	2.2 ± 1.4	1.8 ± 1.3	1.9 ± 1.3	<0.001

Total study population n = 2861, *NGM* Normal glucose metabolism, *T2DM* Type 2 diabetes mellitus (newly or previously diagnosed)

^1^ *p*–values were obtained from ANOVA (p for trend)/ Kruskal-Wallis/ Chi-Square tests

^2^low education (no education, primary education, and lower vocational education). ^3^ intermediate education (intermediate vocational education, higher secondary education, and vocational education). ^4^ high education (higher professional education, university)

^a^Social support variables have a range from 0 to 5. Values are means (SD), unless stated otherwise

### Description of structural characteristics of the social network

**Fig. 1:**
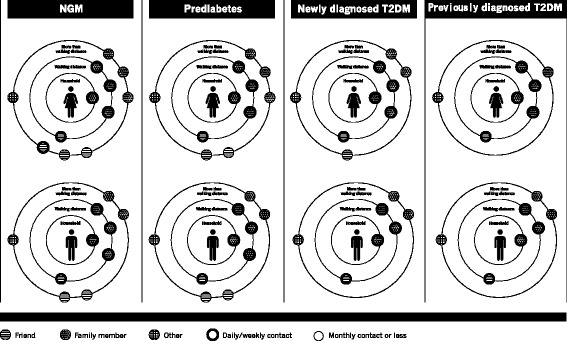
Structural network characteristics stratified by diabetes status among women and men

Figure 1 shows a simplified representation of the social network size, contact frequency, geographic distance, and proportions of family members and friends according to diabetes status for both men and women. In summary, the network size was 12, 11, 9, and 8 in women with NGM, pre-diabetes, newly diagnosed T2DM and previously diagnosed T2DM, respectively. In men, the network size was 10 in NGM and pre-diabetes and 7 in newly diagnosed and previously diagnosed T2DM. The total number of contacts per half year was 268, 252, 224, 212 in women with NGM, pre-diabetes, newly diagnosed T2DM and previously diagnosed T2DM, respectively, and 224, 216, 175, 189 for men, respectively. The percentage of daily/weekly contacts was 46.3% in NGM and 54.2% in previously diagnosed T2DM. The percentage of family members was 55.9% in the NGM group and 64.7% in the previously diagnosed T2DM group. The percentage of friends was 30.0% in NGM and 21.4% in previously diagnosed T2DM (Table 2).

The prevalence of living alone was 14.7%, 17.4%, 17.1% and 20.2% and the prevalence of social participation was 71.6%, 64.2%, 61.1%, and 56.4% in NGM, pre-diabetes, newly diagnosed T2DM and previously diagnosed T2DM, respectively (Table 2).

### Description of functional characteristics of the social network

Participants with newly diagnosed and previously diagnosed T2DM reported lower levels of informational support related to advice on problems, emotional support related to discomfort and related to important decisions and practical support related to jobs around the house and related to sickness than participants with NGM or pre-diabetes (Table 2).

### Association of structural characteristics of the social network with diabetes status

Table 3 shows that each fewer network member reported (smaller network size) was associated with 12% higher odds of newly diagnosed T2DM and a 8% higher odds of previously diagnosed T2DM in women and an 10% and 5% higher odds of newly diagnosed T2DM and previously diagnosed T2DM in men, respectively, compared to NGM. Each 10% drop in network members living within walking distance was associated with an 21% higher odds of newly diagnosed T2DM and with an 9% higher odds of previously diagnosed T2DM in women. Every additional 10% of the network that was a household member was associated with a 25% higher odds of newly diagnosed T2DM and an 15% higher odds of previously diagnosed T2DM in women and a 29% higher odds of newly diagnosed T2DM in men. Each 10% drop in network members who were friends was associated with a 14% higher odds of previously diagnosed T2DM in women.

**Table 3:** Associations of social network characteristics with diabetes status stratified by sex

	*Outcome variables stratified by sex*					
	Women			Men		
Reference category; NGM	Pre-diabetes (*n* = 201)	Newly diagnosed T2DM (*n* = 41)	Previously diagnosed T2DM (*n* = 213)	Pre-diabetes (*n* = 229)	Newly diagnosed T2DM (*n* = 70)	Previously diagnosed T2DM (*n* = 484)
	OR (95% CI)	OR (95% CI)	OR (95% CI)	OR (95% CI)	OR (95% CI)	OR (95% CI)
*Explanatory variables*						
Structural characteristics of the social network						
Smaller network size (for every fewer network member)	1.02 (0.99–1.06)	1.12** (1.03–1.22)	1.08*** (1.04–1.13)	0.99 (0.95–1.02)	1.10** `(1.03–1.18)	1.05** (1.02–1.09)
*Contact frequency*						
Total contacts per half year (for every 10 additional contacts)	1.00 (0.99–1.01)	0.98 (0.96–1.01)	0.98* (0.97–1.00)	1.00 (0.99–1.01)	0.98# (0.96–1.00)	0.99(0.98–1.02)
Percentage of daily-weekly contact (for every additional 10%)	0.99 (0.92–1.05)	1.10 (0.97–1.26)	1.07# (0.99–1.15)	0.99 (0.93–1.05)	1.08# (0.98–1.19)	1.04(0.98–1.09)
*Proximity*						
Percentage of network members living within walking distance (for every fewer 10%)	1.03 (0.95–1.11)	1.21* (1.02–1.42)	1.09* (1.01–1.19)	0.98 (0.91–1.05)	1.02 (0.91–1.13)	1.05# (0.99–1.12)
*Type of relationship*						
Percentage household members (for every additional 10%)	1.06 (0.93–1.20)	1.25** (1.05–1.50)	1.15* (1.03–1.29)	0.96 (0.85–1.08)	1.29*** (1.12–1.49)	0.99 (0.90–1.09)
Percentage family members (for every additional 10%)	1.02 (0.94–1.10)	1.06 (0.92–1.22)	1.08# (0.99–1.17)	0.98 (0.92–1.04)	1.04 (0.94–1.16)	1.03(0.97–1.09)
Percentage friends (for every 10% less)	1.05 (0.96–1.14)	1.14 (0.96–1.35)	1.14** (1.04–1.26)	1.00 (0.93–1.08)	1.08 (0.95–1.22)	1.04(0.98–1.11)
Living alone	1.00 (0.66–1.52)	0.59 (0.24–1.44)	0.87 (0.54–1.39)	1.59# (0.98–2.60)	1.84# (0.89–3.81)	1.94**(1.29–2.93)
Lack of social participation	1.60** (1.12–2.27)	1.72 (0.84–3.55)	2.12*** (1.44–3.13)	1.31 (0.93–1.85)	1.57# (0.92–2.68)	1.42* (1.06–1.90)
Functional characteristics of the social network						
Less informational support^a^	0.98 (0.88–1.10)	1.13 (0.92–1.40)	1.09 (0.97–1.23)	1.02 (0.92–1.12)	1.12 (0.96–1.31)	1.02 (0.93–1.10)
Less emotional support (discomfort) ^a^	1.04 (0.94–1.16)	1.22# (0.97–1.53)	1.12# (0.99–1.27)	1.08 (0.98–1.21)	1.17# (0.98–1.41)	1.06 (0.96–1.16)
Less emotional support (important decisions) ^a^	1.08 (0.96–1.21)	1.34* (1.06–1.69)	1.11# (0.98–1.26)	1.06 (0.95–1.18)	1.19* (1.00–1.43)	1.11* (1.01–1.22)
Less practical support (jobs)^a^	1.11# (1.00–1.24)	1.19 (0.94–1.50)	1.16* (1.02–1.32)	1.03 (0.93–1.15)	1.21* (1.01–1.46)	1.04 (0.95–1.14)
Less practical support (sickness) ^a^	1.07 (0.95–1.20)	1.45* (1.07–1.96)	1.21* (1.05–1.41)	1.08 (0.96–1.21)	1.25* (1.02–1.54)	1.13* (1.02–1.25)

All analyses were adjusted for age, body mass index, educational level, employment status, alcohol consumption, smoking status, Hypertension, prior CVD and general health (SF36). *NGM* Normal glucose metabolism; *T2DM* Type 2 diabetes mellitus. ^**a**^Social support variables have a range from 0 to 5. OR; Odds ratio, 95% CI; 95% Confidence interval. #*p* ≤ 0.1 *p ≤ 0.05 **p ≤ 0.01 ***p ≤ 0.001

**Fig. 2:**
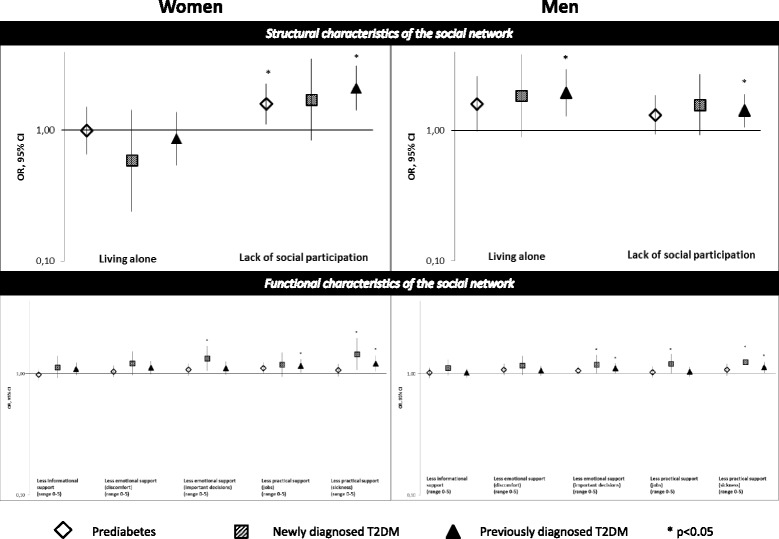
Associations of structural and functional characteristics of the social network with diabetes status stratified by sex, presented on a base-10 logarithmic scale

In women, no significant associations between living alone and diabetes were observed. In men, living alone was associated with a 59% higher odds of pre-diabetes (borderline significant), a 84% higher odds of newly diagnosed T2DM (borderline significant), and a 94% higher odds of previously diagnosed T2DM compared to NGM (Table 3). A lack of social participation was associated with a 60% higher odds of pre-diabetes and a 112% higher odds of previously diagnosed T2DM in women, compared to NGM (Table 3). In men, lack of social participation was associated with a 42% higher odds of having previously diagnosed T2DM. In Fig. 2, ORs for social participation and living alone were depicted.

### Association of functional characteristics of the social network with diabetes status

One unit less emotional support on important decisions was associated with a 34% higher odds of newly diagnosed T2DM in women. One unit less practical support with small jobs was associated with a 16% higher odds of previously diagnosed T2DM in women. One unit less practical support with sickness was associated with a 45% higher odds of newly diagnosed T2DM and a 21% higher odds of previously diagnosed T2DM in women, compared to NGM. In men, one unit less emotional support on important decisions was associated with a 19% higher odds of newly diagnosed T2DM and a 11% higher odds of previously diagnosed T2DM. One unit less practical support with small jobs was associated with a 21% higher odds of newly diagnosed T2DM in men. One unit less practical support with sickness was associated with a 25% higher odds of newly diagnosed T2DM and a 13% higher odds of previously diagnosed T2DM in men, compared to NGM.

## Discussion

This study is the first to assess the associations between T2DM and a broad range of functional and structural network characteristics in adults. The study shows that more socially isolated individuals (smaller social network size) more frequently had newly diagnosed and previously diagnosed T2DM, while this association was not observed with pre-diabetes. In women, proximity and the type of relationship was associated with newly diagnosed and previously diagnosed T2DM. A lack of social participation was associated with pre-diabetes as well as with previously diagnosed T2DM in women, and with previously diagnosed T2DM in men. Living alone was associated with higher odds of previously diagnosed T2DM in men, but not in women. Less emotional support related to important decisions was associated with newly diagnosed T2DM in women, and both newly and previously diagnosed T2DM in men. Less practical support related to jobs was associated with previously diagnosed T2DM in women and newly diagnosed T2DM in men. Less practical support for sickness was associated with newly diagnosed and previously diagnosed T2DM in men and women. These associations were not observed in pre-diabetes.

All associations between social network characteristics and diabetes status were independent of BMI, educational level, employment status, alcohol consumption, smoking status, general health status and chronic conditions as prior CVD and hypertension.

### Structural social network characteristics

The present study showed that social isolation, indicated by a smaller social network size, was associated with higher odds of newly diagnosed and previously diagnosed T2DM in men and women. This finding is in line with longitudinal analyses conducted by Altevers et al. (2015), and Lukaschek et al. (2017) who found that poor structural support (measured by Social Network index [SNI], including a measure of social network size) increased the risk of T2DM [9, 27]. In addition, our data show that a smaller social network size was only associated with T2DM, not with pre-diabetes. This is also consistent with longitudinal data, which did not find significant associations of social integration, including structural characteristics, with pre-diabetes [12]. Furthermore, we as well as Gallo et al. (2015) observed associations between structural network characteristics and T2DM among both sexes [13], while Altevers et al. (2015) found this association among men, but not among women [9]. A possible explanation for this discrepancy is that Altevers et al. (2015) limited the variability in their sample by dichotomizing the Social Network Index (SNI), while we and Gallo et al. (2015) used a continuous scale. Therefore, their non-significant findings in women may be attributable to low power [9].

In women, higher percentages of network members living within walking distance and higher percentages household members were associated with newly and previously diagnosed T2DM. Similarly, a network composed of fewer friends was associated with higher odds of previously diagnosed T2DM in women, suggesting that the smaller network size in T2DM is largely attributable to having less friends than those with NGM. The associations of proximity and the type of relationship with T2DM in women indicate that a network that is centralized to those with the closest relationships, with less network members at a social and geographical distance, is associated with T2DM. In men, we observed that higher percentages of household members were associated with newly diagnosed T2DM. Furthermore, these associations were again not observed in pre-diabetes.

As we are the first to address the composition of the social network in terms of proximity and type of relationship in relation to T2DM, and as significant associations have mostly been observed for women, further research is needed to corroborate our findings.

Living alone was associated with higher odds of newly diagnosed and previously diagnosed T2DM in men, but not in women. This finding is consistent with previous longitudinal studies that identified living alone as a risk factor for T2DM [11, 27], while having a partner decreases the risk for T2DM [12] in men but not in women. Moreover, similar to Hilding et al. (2015), we only found borderline significant associations between living alone and pre-diabetes [12]. However, these non-significant risk estimates may be attributable to a low power, as we had a relatively small sample to address this association (less than 40 men with pre-diabetes were living alone).

The lack of social participation was associated with pre-diabetes in women and with previously diagnosed T2DM in both men and women. In longitudinal research, participation in social activities has been shown to decrease the risk of pre-diabetes and T2DM in women and the risk of pre-diabetes in men [12]. However, in this cross-sectional study, we cannot exclude the possibility that early changes in glucose metabolism may cause non-specific complaints such as tiredness and feeling unwell, which may explain why individuals chose to limit their social participation. In either scenario, social participation may serve as a target for intervention or an indicator suitable for diabetes prevention strategies.

### Functional social network characteristics

In the present study, we observed that less emotional support with important decisions was associated with newly diagnosed T2DM in women, and both newly and previously diagnosed T2DM in men. Less practical support with small jobs was associated with previously diagnosed T2DM in women and newly diagnosed T2DM in men. Less practical support for sickness was associated with newly diagnosed and previously diagnosed T2DM in men and women. Both Norberg et al. (2007) and Jones et al. (2015) showed that low emotional support was associated with T2DM in women [7] and older adults [8], although their methods used to assess functional support were less detailed. The longitudinal results from Norberg et al. (2007) suggest that low functional support increases the risk of T2DM [7].

To our knowledge, this study is the first to assess the association of a broad range of functional support measures with pre-diabetes, newly diagnosed T2DM and previously diagnosed T2DM. Our results indicate that emotional support in important decisions, and practical support with small jobs and in sickness were important characteristics that should be addressed in T2DM prevention strategies. However, in this cross-sectional study, we cannot assess whether participants received an absolutely lower level of functional support, or whether they perceive it as less adequate to their needs (that means relatively lower), and therefore, their satisfaction with functional support is lower. Recently, it has been shown that low social network satisfaction is associated with increased risk of T2DM [27].

### Strengths & Limitations

A major strength of the current study was the measurement of structural and functional characteristics with the use of a name generator, one of the best known, most detailed and most widely used instruments to examine ego-centered network data [28]. This resulted in a much broader range of structural and functional social network characteristics than assessed in previous studies. Next, we were able to examine the associations of structural and functional network characteristics in individuals with pre-diabetes, newly diagnosed and previously diagnosed T2DM compared to those with NGM. The associations of pre-diabetes and newly diagnosed T2DM have rarely been studied before. Moreover, we adjusted the analyses for several different variables, i.e. age, body mass index, educational level, employment status, smoking status, alcohol consumption, general health and chronic medical conditions, showing robust results, which makes residual confounding unlikely. Finally, the population-based design of The Maastricht Study and its size were key assets [22].

A few limitations should also be mentioned. The study is cross-sectional in nature, and therefore, the possibility of reverse causality cannot be excluded. Furthermore, as we performed multiple statistical tests, our analyses may include false positive results. However, the majority of significant associations had a *p*-value ≤0.01 or even ≤0.001, limiting the chance of false positive findings. Additionally, the present study population consisted of relatively healthy participants, as is common in population-based cohort studies, and it is possible that we did not include those in the population who were the most socially isolated. Therefore, we may have underestimated the effect sizes.

### Implications

Targeting social network characteristics may prove a promising prevention strategy for T2DM. More socially isolated individuals (smaller network size) more often had T2DM. Broadening their network should be encouraged, as we have shown that a smaller social network size was associated with T2DM in both men and women. Moreover, social participation was associated with pre-diabetes and previously diagnosed T2DM, stimulating participants to became members of a club may also be considered in future intervention development. In addition, social participation may be used as an indicator in diabetes prevention strategies. Moreover, interventions aiming to generate behavioral change (e.g., physical activity) may also tailor to the social network of the participant, as it has been shown that network targeting can be used to increase the adoption of specific public health interventions [17]. In addition, as men living alone seem to be at a higher risk for the development of T2DM, they should be indicated as high-risk group.

Moreover, targeting social network characteristics may also have benefits for other chronic conditions, as it has been shown that most of those with a long-term disorder are multimorbid [3], and social network characteristics have been found to associate with cardiovascular, endocrine, and immune function [29]. In addition, social isolation and living alone have been found to increase the likelihood of mortality [30].

## Conclusions

To conclude, this study was the first to assess a broad range of structural and functional social network characteristics and their associations with normal glucose metabolism, pre-diabetes, newly diagnosed T2DM and previously diagnosed T2DM in a large sample of 40- to 75-year-old adults. These results were independent of BMI, educational level, employment status, alcohol consumption, smoking status, general health status and chronic conditions as prior CVD and hypertension. Men and women who were more socially isolated, and who received less emotional and practical support, more frequently had newly and previously diagnosed T2DM, while this was not observed in individuals with pre-diabetes. In women, proximity and the type of relationship was associated with newly and previously diagnosed T2DM. A lack of social participation was associated with pre-diabetes in women, as well as with previously diagnosed T2DM in both sexes. Living alone was associated with higher odds of previously diagnosed T2DM in men, but not in women. This study shows that several aspects of structural and functional characteristics of the social network were associated with newly and previously diagnosed T2DM, partially different for men and women. These results may provide useful targets for T2DM prevention efforts.

## Additional file

Additional file 1: A detailed description of the social network questionnaire and the structural characteristics of the social network. Includes a detailed description of the social network questionnaire used in the present report. (DOCX 18 kb)

## Abbreviations

T2DM: Type 2 diabetes mellitus
