# Supplementary material for: Socially isolated individuals are more prone to have newly diagnosed and prevalent type 2 diabetes mellitus - the Maastricht study –
Source: BMC Public Health. 2017 Dec 19;17:955. doi: 10.1186/s12889-017-4948-6 (PMC5735891; doi:10.1186/s12889-017-4948-6)
Supplement: Additional file 1: — A detailed description of the social network questionnaire and the structural characteristics of the social network. Includes a detailed description of the social network questionnaire used in the present report. (DOCX 18 kb) [file 12889_2017_4948_MOESM1_ESM.docx]

**Additional file 1.** A detailed description of the social network questionnaire.

1. **Social network questionnaire**

The participants received a questionnaire with seven questions on different types of contacts and were asked to name a maximum of five persons (network members). The questions concerned 1) persons who advised them on problems, 2) persons who could offer them practical help if they were sick, 3) persons who provided emotional support when they were feeling unwell, 4) persons who helped them with small and larger jobs around the house, 5) persons they visited for social purposes or with whom they could sometimes get together, and 6) persons with whom they could discuss important matters, and finally, 7) the participants were asked to name a maximum number of ten additional persons who were also important to them. In total, participants could name a maximum number of 40 network members. After every question and for each network member named, they were asked to indicate their frequency of contact with this person over the last six months (daily or weekly, monthly, quarterly, and half-yearly). This was asked for all seven types of contacts. Moreover, the participants were asked to identify their relationship to this person (e.g., partner, sister, friend, neighbor, etc. (28 options)), how far away this person lived (walking distance, less than half an hour away by car, more than half an hour away by car, more distant) and to indicate this person’s sex and actual or estimated age.

The participants were also asked whether they were a member of a club (yes/no) and, if so, to identify the club(s) (sports club, religious group, volunteer organization, discussion group, self-support group, Internet club, or another organization) and how often they frequented it (daily/weekly, monthly, occasionally).
